# Deoxynivalenol-Induced Spleen Toxicity in Mice: Inflammation, Endoplasmic Reticulum Stress, Macrophage Polarization, and the Dysregulation of LncRNA Expression

**DOI:** 10.3390/toxins16100432

**Published:** 2024-10-09

**Authors:** Qingbo Zhao, Weili Feng, Peiyu Gao, Yu Han, Siyi Zhang, Ao Zhou, Liangyu Shi, Jing Zhang

**Affiliations:** Laboratory of Genetic Breeding, Reproduction and Precision Livestock Farming & Hubei Provincial Center of Technology Innovation for Domestic Animal Breeding, School of Animal Science and Nutritional Engineering, Wuhan Polytechnic University, Wuhan 430023, China; zhqb0416@126.com (Q.Z.); fengweili0079@126.com (W.F.); gao15846639577@126.com (P.G.); hy13018038681@126.com (Y.H.); m15007191047@126.com (S.Z.); zhouao2008@aliyun.com (A.Z.); liangyu_shi@whpu.edu.cn (L.S.)

**Keywords:** deoxynivalenol, spleen, inflammation, endoplasmic reticulum stress, macrophages, lncRNAs

## Abstract

The spleen is a primary target of deoxynivalenol (DON) toxicity, but its underlying molecular mechanisms remain unclear. This study investigates the effects of DON on inflammation, splenic macrophage polarization, endoplasmic reticulum (ER) stress, and transcriptome changes (mRNA and lncRNAs) in mouse spleen. We found that DON exposure at doses of 2.5 or 5 mg/kg BW significantly induced inflammation and polarized splenic macrophages towards the M1 phenotype. Additionally, DON activated PERK-eIF2α-ATF4-mediated ER stress and upregulated apoptosis-related proteins (caspase-12, caspase-3). The ER stress inhibitor, 4-Phenylbutyric acid, significantly alleviated DON-induced ER stress, apoptosis, and the M1 polarization of splenic macrophages. Transcriptome analysis identified 1968 differentially expressed (DE) lncRNAs and 2664 DE mRNAs in mouse spleen following DON exposure. Functional enrichment analysis indicated that the upregulated genes were involved in pathways associated with immunity, including Th17 cell differentiation, TNF signaling, and IL-17 signaling, while downregulated mRNAs were linked to cell survival and growth pathways. Furthermore, 370 DE lncRNAs were predicted to target 255 DE target genes associated with immune processes, including the innate immune response, interferon-beta response, cytokine production regulation, leukocyte apoptosis, and NF-κB signaling genes. This study provides new insights into the mechanisms underlying DON toxicity and its effects on the immune system.

## 1. Introduction

Deoxynivalenol (DON) is a mycotoxin generated by Fusarium fungi, frequently found in contaminated cereal grains and various animal-derived and processed food products [1]. Its stability during storage and processing makes it difficult to eliminate, resulting in significant contamination and potential toxicity, thus representing a significant risk to worldwide food safety and the health of livestock [2,3].

DON toxicity primarily affects the immune system, thereby impacting the well-being of humans and animals. The spleen is a crucial peripheral immune organ in which immune cells, including lymphocytes and macrophages, encounter pathogens and antigens to initiate innate and adaptive immune responses [4]. DON exposure leads to a reduced spleen index, splenic tissue damage, decreased antioxidant capacity, and increased DNA damage [5]. Recent studies have demonstrated that DON induces oxidative injury, inflammation, apoptosis, and mitochondrial autophagy in splenic lymphocytes [6,7,8]. Macrophages, a principal cell type in the spleen, regulate immune–inflammatory responses by releasing various inflammatory cytokines [9]. Due to their high plasticity, macrophages can polarize into pro-inflammatory M1 or anti-inflammatory M2 phenotypes. An imbalance in M1/M2 polarization resulting in a pro-inflammatory phenotype is implicated in spleen damage [10]. Although studies have indicated that DON has immunotoxicity, research on its effects on the polarization of splenic macrophages is limited.

Endoplasmic reticulum (ER) stress, triggered by various endogenous and exogenous stimuli, leads to incorrectly folded proteins accumulating within the ER lumen, activating the unfolded protein response (UPR) to restore homeostasis and counteract adverse conditions [11]. Prolonged or intense ER stress results in cellular or tissue damage through the activation of ER-stress-associated pathways. The protein kinase RNA-like endoplasmic reticulum kinase (PERK) pathway of the UPR is a component of a conserved intracellular signaling network known as the integrated stress response, which is activated by nutrient deprivation, pathogen infection, and oxidative stress [12]. Recent studies have demonstrated a notable connection between the PERK pathway and mitochondrial dysfunction and apoptosis in the spleen [13,14,15]. Additionally, ER stress regulates macrophage polarization through the PERK pathway [16]. However, it remains unclear whether the PERK pathway participates in DON-induced inflammation and the polarization of splenic macrophages.

Recent evidence indicates that long non-coding RNAs (lncRNAs) contribute to the mechanisms underlying mycotoxin toxicity, acting as potential targets or initiators of toxic effects [17,18]. These lncRNAs can influence gene expression through multiple mechanisms, such as modifying chromatin, regulating transcription, and impacting post-transcriptional processing, which in turn influences the immune response and inflammation [19]. A study has shown that LncRNA Gm20319 is involved in DON-induced liver damage [20]. Although lncRNAs have been demonstrated to be significant in modulating host immune and inflammatory responses to pathogen infections or toxins, their functional importance in DON-induced spleen injury is still unclear.

In this study, we utilized C57BL/6 mice as the experimental model and administered DON (2.5 or 5 mg/kg body weight) via injection for 3 h to assess inflammation, ER stress and spleen macrophage polarization. Utilizing high-throughput transcriptome sequencing, we identified immune-related lncRNAs and mRNAs in the spleens of mice subjected to DON treatment. This study aims to clarify the roles of ER stress and immune-related lncRNAs in DON toxicity, providing novel perspectives on the molecular mechanisms driving DON-induced splenic damage.

## 2. Results

### 2.1. DON-Induced Inflammation in Mouse Spleen

**Figure 1:**
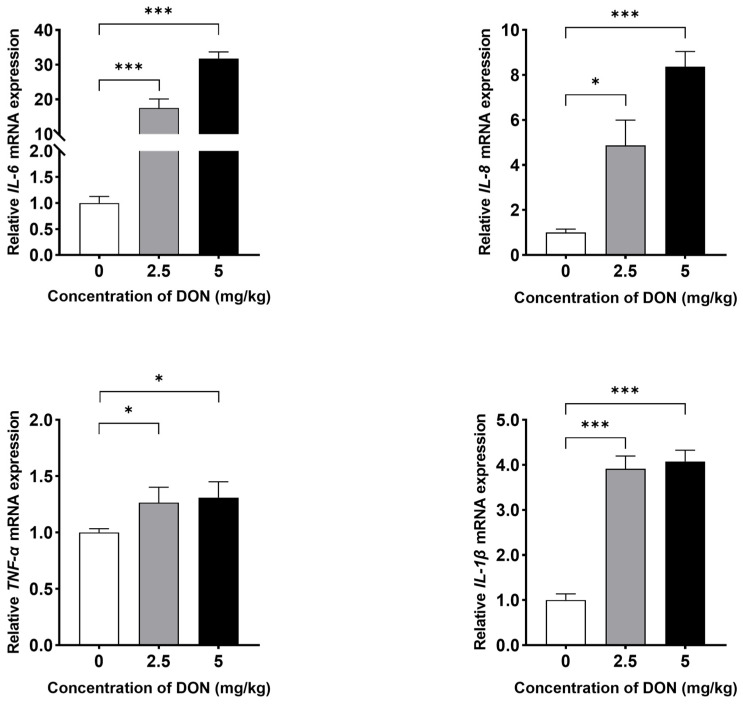
The impacts of DON on inflammation in the spleen of mice. Mice were administered phosphate-buffered saline (PBS) or DON at doses of 2.5 or 5 mg/kg BW for 3 h. The expressions of IL-6, IL-8, TNF-α, and IL-1β were measured in the spleen of three different groups. Data were expressed as mean ± SD (n = 4), with untreated mice (0 μM) serving as the control. Statistical significance was denoted as follows: * *p* < 0.05 and *** *p* < 0.001.

Compared to the control group, DON treatment triggered inflammation in the spleens of mice. As illustrated in Figure 1, stimulation with DON at doses of 2.5 or 5 mg/kg BW significantly increased IL-6, IL-8, IL-1β, and TNFα expressions (*p* < 0.05), indicating a dose-dependent inflammatory response in mouse spleen.

### 2.2. DON Stimulation Resulted in the M1 Polarization of Splenic Macrophages

**Figure 2:**
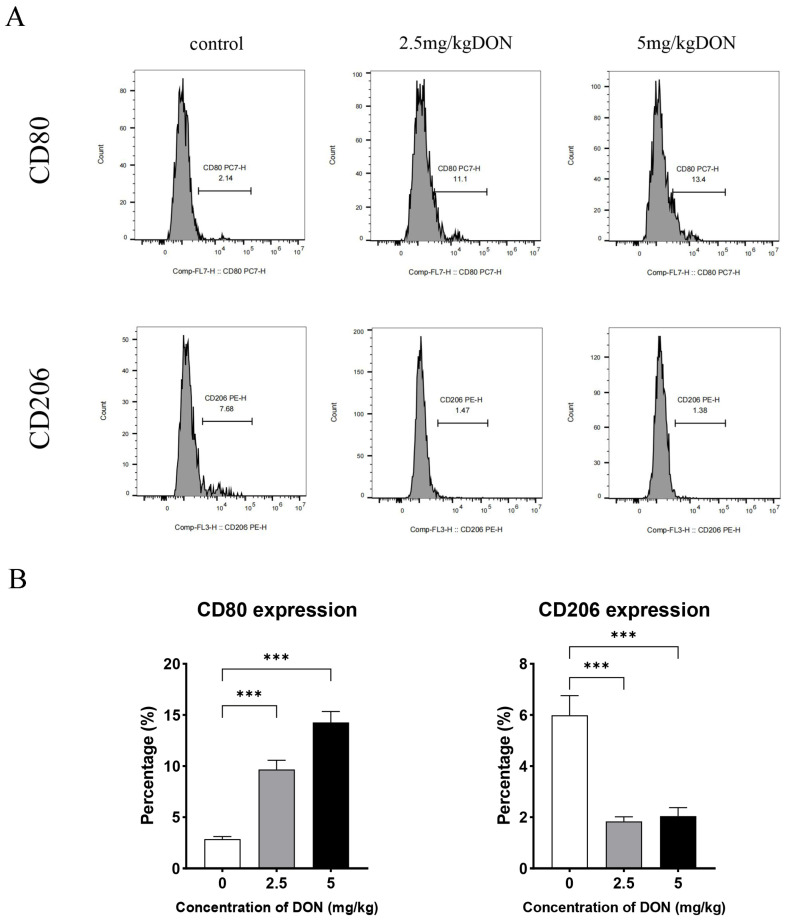
Flow cytometry was employed to evaluate macrophage polarization in the spleen (**A**). The proportions of M1 (CD80+) and M2 (CD206+) macrophage cell populations were calculated (**B**). Data were expressed as mean ± SD (n = 4). Statistical significance was denoted as follows: *** *p* < 0.001.

The phenotypic switch in macrophage polarization in the spleens of DON-treated mice was analyzed using flow cytometry. As shown in Figure 2, DON significantly increased the proportions of M1 macrophages (CD80-positive) in a dose-dependent manner, while noticeably decreasing the proportion of M2 macrophages (CD206-positive) in the total splenic macrophage population. These findings suggest that DON stimulation induces the polarization of mouse splenic macrophages to the M1 phenotype.

### 2.3. DON Stimulation Triggered the Activation of the PERK Pathway in the Mouse Spleen

**Figure 3:**
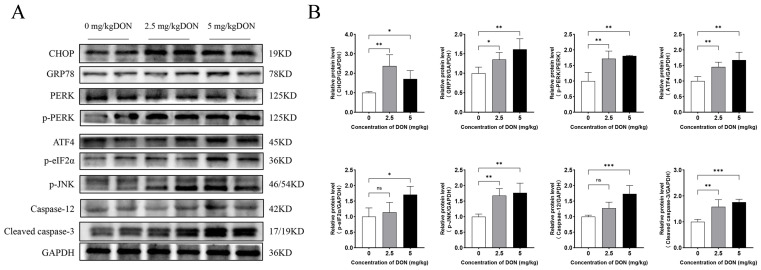
The impact of DON on the expression of PERK/ATF4 pathway-related genes in the mouse spleen was examined. (**A**) Protein expression levels of CHOP, GRP78, PERK, p-PERK, ATF4, p-eIF2α, p-JNK, Caspase-12, and cleaved caspase-3 were assessed in the spleens of mice treated with DON at either 2.5 or 5 mg/kg BW for 3 h. (**B**) A statistical analysis graph depicting these protein expression levels was generated using ImageJ software (ver1.48). Data were expressed as mean ± SD (n = 4). Statistical significance was denoted as follows: * *p* < 0.05, ** *p* < 0.01, *** *p* < 0.001. ns = not significant.

As illustrated in Figure 3, several classic markers of the PERK/ATF4 pathway of the UPR were detected, including CHOP, GRP78, PERK, p-PERK, ATF4, p-eIF2α, p-JNK, and Caspase-12. DON at doses of 2.5 or 5 mg/kg BW significantly increased the protein expression levels of CHOP, GRP78, ATF4, p-JNK, and p-PERK in mouse spleen compared to the control group (*p* < 0.05). However, the expressions of p-eIF2α and Caspase-12 were only significantly enhanced at the dose of 5 mg/kg BW (*p* < 0.05). Additionally, DON at both doses significantly elevated cleaved caspase-3 expression, a key marker of apoptosis, in mouse spleen.

### 2.4. 4-Phenylbutyric Acid (4-PBA) Alleviated PERK Pathway Activation and M1 Polarization in Splenic Macrophages

**Figure 4:**
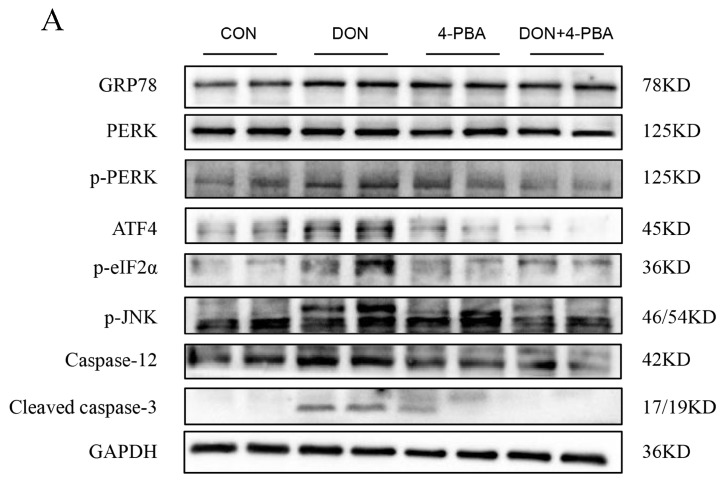

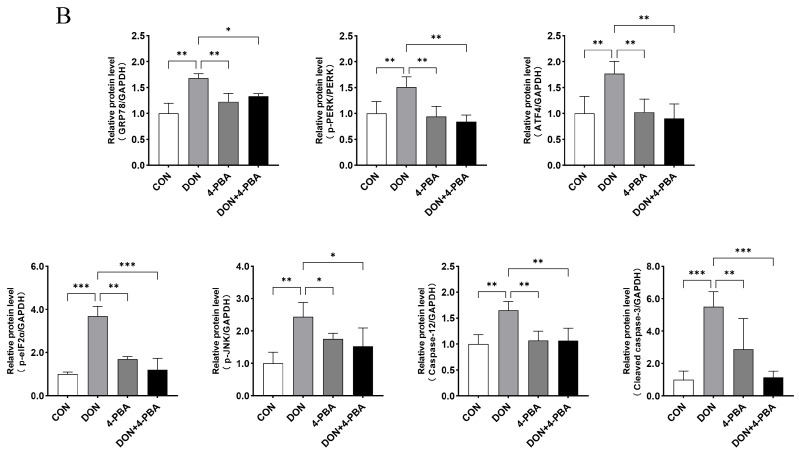
The impact of 4-PBA on the PERK/ATF4 pathway in DON-treated mice was evaluated. (**A**) Protein expression levels of GRP78, PERK, p-PERK, ATF4, p-eIF2α, p-JNK, Caspase-12, and cleaved caspase-3 in the spleens of mice under various treatment conditions were assessed. (**B**) A statistical analysis graph illustrating these protein expression levels was created using ImageJ software. Data were expressed as mean ± SD (n = 4). Statistical significance was denoted as follows: * *p* < 0.05,** *p* < 0.01, and *** *p* < 0.001.

**Figure 5:**
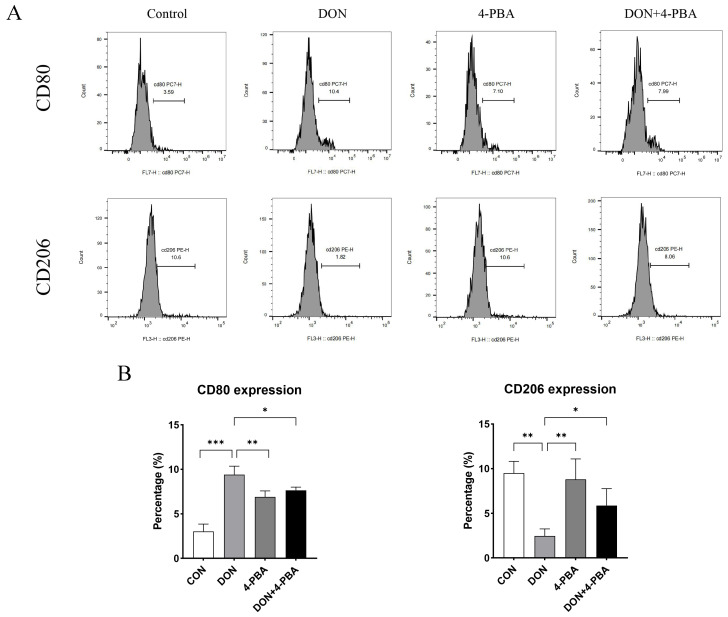
The effect of 4-PBA on the DON-induced polarization of splenic macrophages was evaluated. (**A**) The detection of splenic macrophage polarization was performed via flow cytometry under various treatment conditions. (**B**) The proportions of M1 (CD80+) and M2 (CD206+) macrophage cell populations were calculated. Data were expressed as mean ± SD (n = 4). Statistical significance was denoted as follows: * *p* < 0.05,** *p* < 0.01, and *** *p* < 0.001.

Figure 4 showed that DON increased the protein levels of GRP78, p-PERK, ATF4, p-eIF2α, p-JNK, Caspase-12, and cleaved Caspase-3 (*p* < 0.05). However, 4-PBA significantly eliminated this effect in mouse spleen (*p* < 0.05). In addition, we evaluated the effect of 4-PBA on the DON-induced polarization of splenic macrophages. The results, as shown in Figure 5, suggest that 4-PBA inhibited DON-induced M1 polarization and promoted M2 polarization.

### 2.5. LncRNA and mRNA Expression Alterations in Mouse Spleen after DON Treatment

To investigate the characteristics of lncRNA changes induced by DON (5 mg/kg BW) in mice, we performed high-throughput sequencing on spleen tissue samples from DON-treated mouse groups (DON 1, 2, 3) and control groups (Control 1, 2, 3). We achieved clean reads with Q30 > 95% in each library, as follows: Control-1 (86,628,642), Control-2 (82,258,962), Control-3 (84,666,070), DON-1 (75,510,548), DON-2 (86,456,360), and DON-3 (81,309,248) (Supplementary Table S1). The six libraries were aligned to the mouse reference genome, with a mapping rate exceeding 96%.

**Figure 6:**
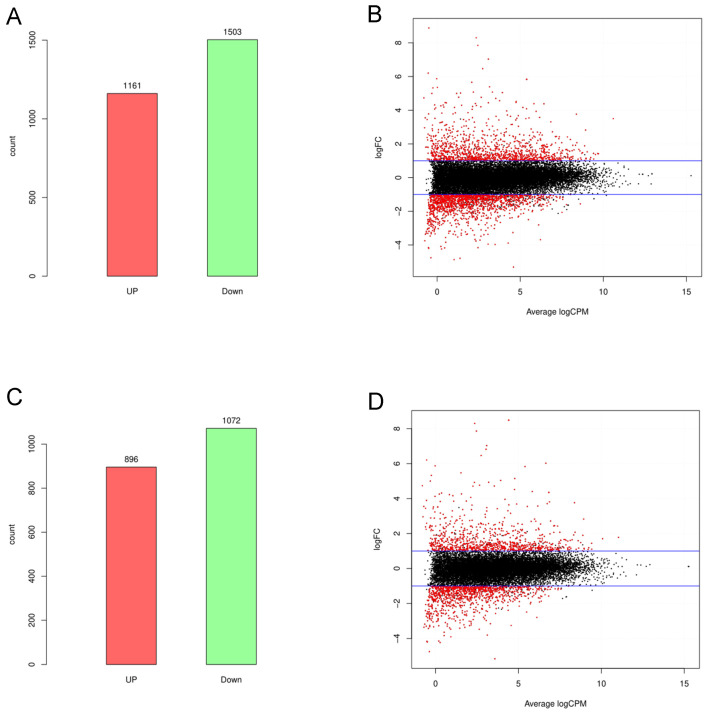
Expression changes in lncRNAs and mRNAs in splenic tissue after DON stimulation, including differentially expressed (DE) mRNAs (**A**) and DE lncRNAs (**C**). MA plot of DE mRNAs (**B**) and DE lncRNAs (**D**).

A total of 2664 differentially expressed (DE) mRNAs were identified in the spleen after DON stimulation, with 1161 upregulated and 1503 downregulated (Figure 6A, Supplementary Table S2). Additionally, 1968 DE lncRNAs were identified, with 896 upregulated and 1072 downregulated (Figure 6C, Supplementary Table S3). The DE mRNAs and DE lncRNAs were visualized using MA plots (Figure 6B,D). In DE lncRNA, we identified 617 novel lncRNAs, which are listed in Supplementary Table S3.

### 2.6. Function and Pathway Enrichment Analysis of DE mRNAs

**Figure 7:**
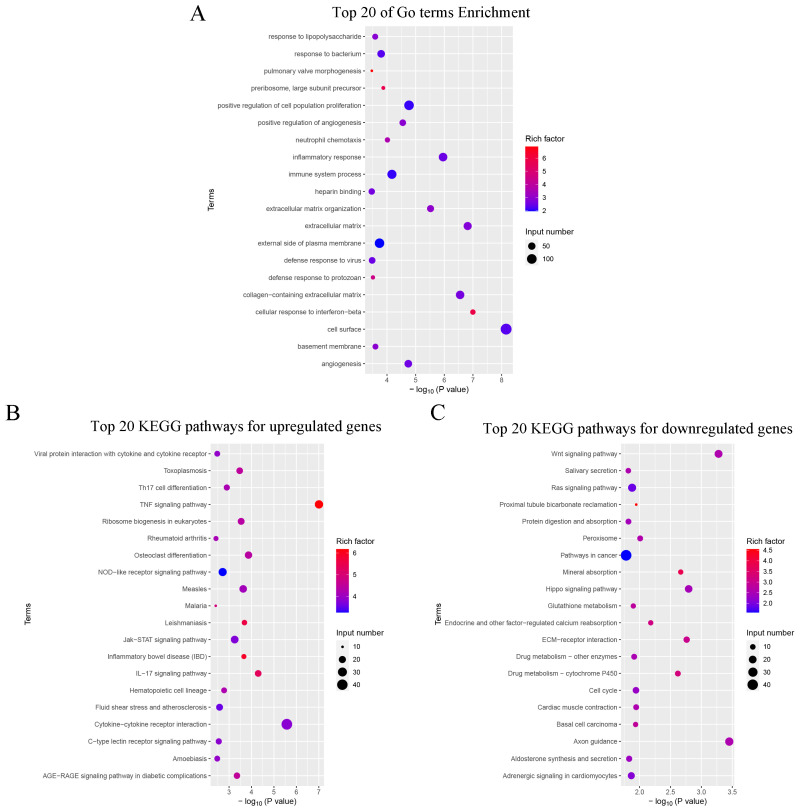
GO and KEGG pathway analyses of the DE mRNAs. (**A**) The top 20 significantly enriched GO terms. (**B**) The top 20 enriched KEGG pathways for upregulated DE mRNAs. (**C**) The top 20 enriched KEGG pathways for downregulated DE mRNAs.

GO functional categorization was conducted to assess the potential functions of DE mRNAs, dividing them into three categories: 49 molecular function (MF) terms, 43 cellular component (CC) terms, and 263 biological process (BP) terms (Supplementary Table S4). Figure 7A displayed the top 20 significantly enriched GO terms related to immune functions, including inflammatory response, immune system processes, and neutrophil chemotaxis. The top 20 enriched pathways for upregulated DE mRNAs are shown in Figure 7B. KEGG analysis showed that the upregulated DE mRNAs were prominently associated with immune-related pathways, including the TNF signaling pathway, Th17 cell differentiation, IL-17 signaling pathway, NOD-like receptor signaling pathway, and cytokine–cytokine receptor interaction. Conversely, KEGG analysis revealed that downregulated DE mRNAs were significantly implicated in pathways associated with cell survival and growth, including the Wnt signaling pathway, Ras signaling pathway, Hippo signaling pathway, and cell cycle (Figure 7C).

### 2.7. Target Gene Prediction of DE lncRNAs

**Figure 8:**
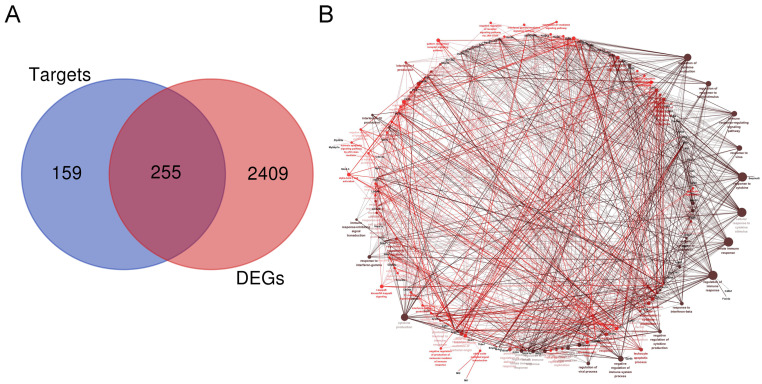
ClueGO enrichment analysis of DETGs. (**A**) Venn diagram illustrating the target genes of DE lncRNAs and DEGs. (**B**) The interaction network of GO BP terms generated by the Cytoscape plug-in ClueGO.

We identified 414 target genes by intersecting the co-expressed genes and cis-targets of DE lncRNAs (Supplementary Table S5). By overlapping the 414 target genes of DE lncRNAs with DE mRNAs, we identified 255 differentially expressed target genes (DETGs) of the 370 DE lncRNAs, as shown in Figure 8A and listed in Supplementary Table S6. Using GlueGO to analyze the function, the results showed that the 255 DETGS were associated with immune system processes, such as the regulation of the innate immune response, response to interferon-beta, regulation of cytokine production, response to cytokine, leukocyte apoptotic process, response to interferon-gamma, IL-10 production, IL-1 production, IκB kinase/NF-κB signaling, and nitric oxide-mediated signal transduction (Figure 8B).

### 2.8. DE lncRNA Verification Using RT-qPCR

**Figure 9:**
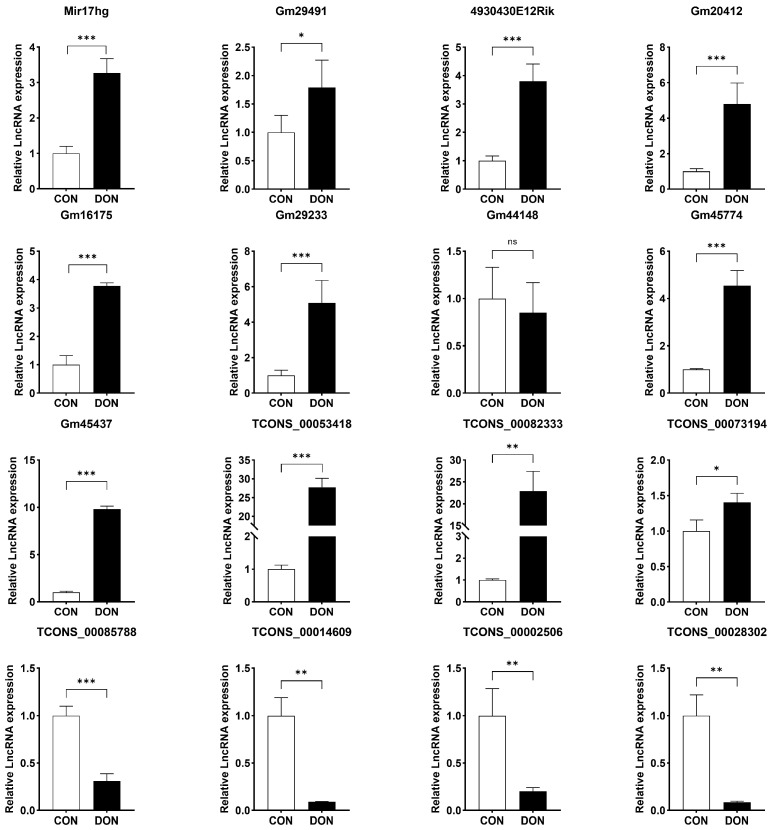
qPCR validation of the sixteen selected lncRNAs in mouse spleens after stimulation with DON (5 mg/kg BW) for 3 h. The data were expressed as mean ± SD (n = 3). Statistical significance was denoted as follows: * *p* < 0.05, ** *p* < 0.01, *** *p* < 0.001 and ns *p* > 0.05.

To validate the sequencing data, we selected sixteen lncRNAs and assessed their expression in mouse spleen under various treatment conditions using RT-qPCR. The expression levels of Mir17hg, Gm29491, 4930430E12Rik, Gm20412, Gm16175, Gm29233, Gm45774, Gm45437, TCONS_00053418, TCONS_00073194, and TCONS_00082333 were significantly upregulated by DON treatment compared to the control group, while TCONS_00002506, TCONS_00085788, TCONS_00014609, and TCONS_00028302 were significantly downregulated (Figure 9). These results were in accordance with the sequencing data, providing further validation for the reliability of our findings.

## 3. Discussion

Low-dose DON (1–5 mg/kg BW) in mice stimulates immune responses by upregulating cytokines and inflammation-related genes, such as TNF-α, IL-1β, and IL-6 [21]. Consistently, in the present study, we treated mice with DON at doses of 2.5 or 5 mg/kg BW for 3 h, which significantly induced IL-6, IL-1β, TNF-α, and IL-8 levels in the spleen. This indicates that low-dose DON-induced acute toxicity triggers spleen inflammation. The macrophage polarization is a critical determinant of the inflammatory response. M1 macrophages drive the pro-inflammatory responses necessary for pathogen clearance and acute inflammation, whereas M2 macrophages facilitate the resolution of inflammation and tissue repair [22,23,24]. Liu et al. illustrated that low doses of DON elevated the levels of IL-6 and TNF-α in piglets and porcine alveolar macrophages (PAMs). Their flow cytometric profiling of specific markers, CD80 (M1-type) and CD206 (M2-type), confirmed that DON exposure induced PAM transformation to the M1 phenotype [25]. Macrophages in the spleen play critical roles in immune function, blood filtration, and the clearance of old or damaged red blood cells [26,27]. In our study, we found that 2.5 or 5 mg/kg BW DON stimulation for 3 h induced the polarization of splenic macrophages towards the M1 phenotype in mice. This demonstrates that DON induces acute inflammation in the spleen, likely associated with M1 macrophage polarization.

There are studies showing that DON exposure triggers ER stress and UPR pathways [28,29,30]. The PERK pathway plays a role in the cellular response to ER stress, which can be implicated in spleen injury [13,14,15]. It activates the PERK phosphorylate, eIF2α, reducing general protein synthesis while selectively upregulating ATF4 translation. ATF4 activates UPR target genes, including CHOP, which promotes apoptosis if ER stress is prolonged or severe [31,32]. In our study, DON stimulation for 3 h significantly elevated the levels of CHOP, p-PERK, ATF4, and p-eIF2α in mouse spleen. Additionally, the ER stress marker GRP78 and the apoptosis marker cleaved caspase-3 were markedly upregulated. As a transcription factor activated by ER stress, CHOP serves as a key marker for ER stress-induced apoptosis [33]. ER stress can also trigger apoptosis via the caspase-12 and JNK pathways [34,35]. Our study revealed that DON raised the levels of p-JNK and caspase-12 in the mouse spleen. As an ER stress inhibitor, 4-PBA decreases ER stress markers and has been demonstrated to attenuate ER-stress-related apoptosis and inflammation in diverse cellular and animal models [36,37,38]. In our study, we employed 4-PBA to investigate the ER stress-associated pathway in DON-treated mouse spleens. Our findings demonstrated that the 4-PBA+DON group showed a significant decrease in the protein levels of GRP78, p-PERK, ATF4, p-eIF2α, p-JNK, caspase-12, and cleaved caspase-3 compared to the DON group, suggesting that DON triggers ER-stress-associated apoptosis in the mouse spleen. Interestingly, we found that 4-PBA inhibited DON-induced M1 polarization and promoted M2 polarization in splenic macrophages. ER stress is activated in macrophages, leading to their polarization towards a pro-inflammatory phenotype [39]. Li et al. found that ascorbic acid 6-palmitate restored the M1/M2 polarization balance in LPS-stimulated BV-2 microglial cells through modulation of PERK/eIF2α-mediated ER stress [40]. Yang et al. reported that palmitic-acid-induced ER stress promotes M1 macrophage polarization, which is inhibited by 4-PBA and the PERK inhibitor GSK2656157. PERK knockdown also shifted macrophages from M1 to M2 by altering the STAT1 and STAT6 pathways [16].

In the present study, we also examined the transcriptomic impact of DON on splenic tissue, identifying 2664 DE mRNAs and 1968 DE lncRNAs, highlighting the extensive influence of DON on both protein-coding and non-coding RNA species. GO analysis revealed a significant enrichment of DEGs in immune-associated processes, such as inflammatory response, immune system processes, and neutrophil chemotaxis, indicating that the immune response in the mouse spleen tissue was significantly enhanced after DON stimulation. Th17 cells, a subset of CD4^+^ T helper cells, express IL-17A, IL-17F, and IL-22 upon activation and are crucial for defending against extracellular pathogen infections [41]. IL-17 signaling promotes the production of pro-inflammatory cytokines (e.g., IL-6, TNF-α), chemokines (e.g., CXCL1, CXCL2), and antimicrobial peptides (e.g., β-defensins), which are essential for neutrophil recruitment and activation [42,43]. Cano et al. found that DON triggered a pathogenic Th17 cell subset in porcine jejunal explants [44]. In this study, we also found that upregulated genes were significantly enriched in the Th17 cell differentiation and IL-17 signaling pathways in the spleen following DON stimulation. However, the downregulated genes were enriched in the Wnt, Ras, and Hippo signaling pathways, which are crucial for regulating cell growth, survival, and differentiation [45,46,47].

In this study, we identified 255 DETGs of 370 dysregulated lncRNAs in the spleen following DON stimulation. The GO functional analysis of these 255 DETGs revealed that the putative functions of these 370 lncRNAs are primarily involved in various immune regulatory biological processes. In this study, we observed a significant upregulation of lncRNAs Gm16175, Gm29233, Gm20412, and Gm29491 in mouse spleen following DON treatment and predicted their target genes to be B Cell Lymphoma-3 (BCL-3), Thrombospondin 1 (THBS1), Transglutaminase 2 (TGM-2), and Transforming growth factor beta 2 (TGFB2), respectively. Bcl-3, part of the atypical IκB family, influences NF-κB activity and is involved in regulating B cell development, Th cell differentiation, survival, and proliferation, with contradictory effects on inflammation [48,49]. THBS1, a matricellular protein, is strongly expressed during inflammation and plays roles in inducing macrophage IL-10 production, inhibiting angiogenesis, modulating immune responses, and affecting tumor development [50,51,52]. TGM-2 is reported to have multiple roles, including facilitating tissue formation, promoting wound healing, regulating the cell cycle, mediating inflammatory responses, enabling fiber formation, and offering protection against infections [53,54,55]. As a TGFB subtype, TGFB2 is produced by neutrophils, eosinophils, macrophages, and regulatory T cells, and it is closely linked to immune responses [56]. Thus, the induction of lncRNAs Gm16175, Gm29233, Gm20412, and Gm29491 in the spleen by DON suggests they may play significant roles in the immune response to DON. However, the specific molecular mechanisms by which lncRNAs in the spleen participate in DON-induced immune regulation require further investigation.

## 4. Conclusions

Our study demonstrates that DON-induced acute toxicity at doses of 2.5 or 5 mg/kg BW triggers inflammation in the mouse spleen and facilitates the polarization of splenic macrophages to the M1 phenotype after 3 h of stimulation. Treatment with 4-PBA markedly diminished the levels of GRP78, p-PERK, ATF4, p-eIF2α, p-JNK, caspase-12, and cleaved caspase-3 in the spleen, highlighting the role of DON in inducing ER stress-associated apoptosis. Additionally, 4-PBA reversed DON-induced M1 polarization and enhanced M2 polarization in splenic macrophages. Moreover, our study provides the first comprehensive description of the lncRNA and mRNA profiles in the mouse spleen following DON stimulation, identifying several DE lncRNAs and DE mRNAs associated with immune regulation. Further investigations are required to evaluate the biological functions of these lncRNAs and their roles in signaling pathways responding to DON. These findings provide a better understanding of the molecular mechanisms underlying DON toxicity and suggest potential therapeutic strategies to mitigate its adverse effects on the immune system.

## 5. Materials and Methods

### 5.1. Experimental Design and Animal Treatment

Aged 5–6 weeks, male C57BL/6 mice were sourced from Hubei Yizhicheng Biotechnology Co., Ltd. (Wuhan, China). The mice were kept at 25 ± 2 °C with a 12 h light–dark cycle and had unlimited access to food and water. DON (HY-N6684) and 4-PBA (HY-A0281) were obtained from MedChemExpress (South Brunswick, NJ, USA). Animal experiments were independently replicated twice. Following a 7-day acclimatization period, the mice were randomly divided into three experimental groups for each trial: the CON group (n = 4), the 2.5 mg/kg BW DON group (n = 4), and the 5 mg/kg BW DON group (n = 4). The respective groups received PBS, 2.5 mg/kg BW DON, or 5 mg/kg BW DON via intraperitoneal injection [57]. After 3 h, the mice were sacrificed to collected spleen tissues.

To investigate the function of ER stress in DON-induced tissue damage, a separate experiment was conducted. Mice were randomly divided into four groups: CON, DON, 4-PBA, and DON+4-PBA (n = 4 per group). Mice received an intraperitoneal injection of 500 mg/kg BW 4-PBA and were subsequently treated with either PBS or 5 mg/kg BW DON [58]. Samples were collected 3 h after DON treatment.

The animal study protocol was approved by the Ethics Committee at Wuhan Polytechnic University, designated as protocol code WPU202301002. All animal procedures were carried out in accordance with the Hubei Provincial Regulation on Laboratory Animal Administration.

### 5.2. Quantitative Real-Time PCR (qRT-PCR)

cDNA synthesis and qRT-PCR were performed following established protocols [32]. We measured gene expression levels using the QuantStudio 1 Plus Real-Time PCR System (Applied Biosystems, Foster City, CA, USA), with GAPDH as the housekeeping gene. The 2^−ΔΔCT^ method was employed for data analysis. Primer sequences are detailed in Supplementary Table S7.

### 5.3. Immunostaining and Flow Cytometry of Spleen Single-Cell Suspensions

The splenic cell suspension was harvested by mechanical grinding and filtration in a 40 μm cell strainer. After obtaining the single-cell suspension, the red blood cells were gently lysed using ammonium chloride, and the spleen single-cell suspension was stained with fluorescently conjugated antibodies against CD11b, CD110, F4/80, CD80, and CD206 (Biolegend, San Diego, CA, USA). After 20 min of 4 °C staining, the cells were washed with FACS buffer (0.5% sodium azide in PBS). Subsequently, we performed flow cytometry analysis (Beckman, Brea, CA, USA) for detection.

### 5.4. Western Blot

Total proteins from spleen tissues were extracted using RIPA lysis buffer (R0278, Sigma-Aldrich, St. Louis, MO, USA) with 2% protease phosphatase inhibitor (P1050, Beyotime, Shanghai, China). Protein concentration was determined using a BCA protein analysis kit (P0010, Beyotime). A 20 μg aliquot of protein was separated by electrophoresis on a polyacrylamide gel and transferred to a PVDF membrane. Membranes were incubated for 2 h in TBST buffer containing 5% skimmed milk powder (GC310001, Servicebio, Wuhan, China) for blocking, after which primary antibodies were incubated overnight at 4 °C. Primary antibodies used: Caspase12 (WL03268), Cleaved-caspase3 (WL01992), CHOP (WL00880), GRP78 (WL03157), ATF4 (WL02330), p-eIF2α (AP0341, Abclonal, Wuhan, China), p-JNK (WL01813), PERK (WL03378), p-PERK (WL05295), and GAPDH (AC001, Abclonal, Massachusetts, UK). The membranes were subsequently incubated with HRP-conjugated secondary antibody (WLA023, Wanleibio, Shenyang, China) at room temperature for 1 h. Protein bands were detected using BeyoECL Star (P0018AS, Beyotime), with GAPDH acting as a loading control. Quantitative analysis was carried out using ImageJ software.

### 5.5. High-Throughput Sequencing

Total RNA extraction from spleen tissue was performed using Trizol (Invitrogen, Waltham, MA, USA). Qualified RNA from Control and DON (5 mg/kg BW) groups (n = 3 per group) was utilized for lncRNA sequencing. The six sequencing libraries were constructed according to the manufacturer’s guidelines and sequenced on a DNBSEQ-T7 sequencer (MGI Tech Co., Ltd., Shenzhen, China) with PE150, performed by Seqhealth Technology Co., Ltd. (Wuhan, China). Low-quality reads and adapters were removed from the raw data, and the resulting high-quality reads were then mapped to the mouse GRCm39 reference genome using STAR software (version 2.5.3a). For lncRNA analysis, new lncRNAs were predicted as follows: Mapped reads were spliced to generate new transcripts using StringTie (version 1.3.2). Transcripts were filtered according to four criteria: (1) Exclude transcripts shorter than 200 bp; (2) Exclude transcripts with fewer than 2 exons; (3) Retain transcripts with at least 3 reads; (4) Remove transcripts identified in the genome annotation file. The coding potential of these transcripts was evaluated using CPC2, CPAT, CNCI, and Pfam. Transcripts identified as non-coding by all four tools were classified as novel lncRNAs.

The expression levels of lncRNAs and mRNAs in the Control and DON groups were normalized using RPKM (Reads Per Kilobase per Million). Significant differentially expressed genes were identified based on the criteria of |log2 Fold Change (FC)| ≥ 1.0 and *p*-value < 0.05.

### 5.6. The Target Gene Prediction of the DE lncRNAs

To explore the potential functions of the *DE* lncRNAs, we predicted cis-target genes within 100 kb upstream and downstream of each lncRNA. Co-expression analysis of mRNA and lncRNA was conducted using WGCNA (version 1.51), maintaining a weight value and a signed R2 scale-free topology model fit above 0.9. We then intersected the cis-target and co-expression results to obtain the final predicted target genes.

### 5.7. Functional Enrichment Analysis

GO and KEGG analyses for DEGs were performed using KOBAS (version 2.1.1), applying a significance threshold of *p* < 0.05.

Functional enrichment of the DETGs was analyzed using ClueGO, a Cytoscape plug-in that visualizes the functional classifications of target genes.

### 5.8. Statistical Analysis

Data are expressed as mean ± standard deviation (SD). Statistical significance was assessed using ANOVA and Student’s paired *t*-test. Analyses were performed with GraphPad Prism (version 9.5), considering *p*-values < 0.05 as significant.

## Data Availability

The raw sequence data reported in this paper have been deposited in the Genome Sequence Archive (Genomics, Proteomics & Bioinformatics 2021) in National Genomics Data Center (Nucleic Acids Res 2022), China National Center for Bioinformation/Beijing Institute of Genomics, Chinese Academy of Sciences (GSA: CRA018191) that are publicly accessible at https://ngdc.cncb.ac.cn/gsa, accessed on 5 August 2024.

## Supplementary Materials

The following supporting information can be downloaded at: https://www.mdpi.com/article/10.3390/toxins16100432/s1, Table S1: Sequencing data from mouse spleen tissue; Table S2: Differentially Expressed mRNAs in the Spleen of DON-Treated Mice; Table S3: Differentially Expressed lncRNAs in the Spleen of DON-Treated Mice; Table S4: Go functional classification of the DE mRNAs; Table S5: The targets of the DE lncRNAs; Table S6: The 255 DETGs of the 370 DE lncRNAs; Table S7: Primer sequence information.
