# Supplementary material for: Deoxynivalenol-Induced Spleen Toxicity in Mice: Inflammation, Endoplasmic Reticulum Stress, Macrophage Polarization, and the Dysregulation of LncRNA Expression
Source: Toxins (Basel). 2024 Oct 9;16(10):432. doi: 10.3390/toxins16100432 (PMC11511314; doi:10.3390/toxins16100432)
Supplement: Supplementary file 1 [file toxins-16-00432-s001.zip › supplematry table S1.pdf]

Table S1. Sequencing data from mouse spleen tissue.

Sample name	Raw reads	Raw Q30（%）	Clean reads	Clean Q30（%）	Mapped reads	Mapped ratio（%）
Control_1	97,603,804	95.40	86,628,642	97.92	83,917,063	96.87
Control_2	91,057,962	95.87	82,258,962	97.90	80,090,114	97.36
Control_3	95,451,578	95.32	84,666,070	97.81	82,614,593	97.58
DON_1	84,085,726	95.78	75,510,548	98.04	73,603,710	97.47
DON_2	95,986,512	95.83	86,456,360	98.01	84,474,125	97.71
DON_3	90,806,030	95.75	81,309,248	98.13	79,241,620	97.46
