# Supplementary material for: Deoxynivalenol-Induced Spleen Toxicity in Mice: Inflammation, Endoplasmic Reticulum Stress, Macrophage Polarization, and the Dysregulation of LncRNA Expression
Source: Toxins (Basel). 2024 Oct 9;16(10):432. doi: 10.3390/toxins16100432 (PMC11511314; doi:10.3390/toxins16100432)
Supplement: Supplementary file 1 [file toxins-16-00432-s001.zip › supplematry table S7.pdf]

Table S7 Primer sequence information

Gene	Forward Primer (5′-3′)	Reverse Primer (5′-3′)
GAPDH	AAGCCCATCACCATCTTCCA	CACCAGTAGACTCCACGACA
IL-6	GCCCACCAAGAACGATAGTC	GTCGTTGTCACCAGCATCAG
IL-8	GGCTTTGCGTTGATTCTGG	CGGTGTCCTGATTATCGTCCT
TNF-α	GCCCCCAGTCTGTATCCTTCTA	TTCGGAAAGCCCATTTGAGT
IL-1β	TGCCACCTTTTGACAGTGATG	TGATGTGCTGCTGCGAGATT
TCONS_00053418	TGACGGCAGCGATTAGTGACA	TGACTCTGGAGCATTGGAAGGA
TCONS_00073194	CACTATGGCTGCTGTTCTTGGT	TGAGGCTTGGCACTGTGAGA
TCONS_00082333	GGAGCGGATTGACCAATGAAG	TTGAGCGTCTAATGGAGGCATA
TCONS_00002506	TCTAGTTCCACTCTTGCCTACC	AGCCTGAAGGAGAATAGACCAA
TCONS_00085788	ACAGACAGACAAGGCAAGCA	CCTGACCTCCATGTGATGTAGT
TCONS_00014609	ATGTCACCTGTATTGCCTGCTA	GCTCTGCTTCACCATCACCTA
TCONS_00028302	CCTCCACTTCGTCCTCCTTC	GCTGCCACTTCTCTTGTTCTC
Mir17hg	ACTTGTTCAGTTCCGCACA	TTAGTAACCCACCCCCAT
Gm29491	CATCACGATTCTGCCCG	TTTTCCACACCCCCTCC
4930430E12Rik	ATTGACCACCTAAAACATCTCC	GGCTAATACATCTTCACCTTCC
Gm20412	CGAGAAATTGTTGGACGG	CAAGGGTAAGAGGAGAGCC
Gm16175	CCAGAATACATCTGAGCGTT	CCGTCCCCTTCACCTAG
Gm29233	GGCTGAGACGCCATCTGTATGC	GCACCTGCGGAATGCACTGT
Gm44148	TGTTTTGAGAAGAGGGTGAGAC	GCAGAAGAGAGGAAGAGAAGTGT
Gm45774	GAAAAGTGGAAACGGAGACGC	GGCAAACACCCTGGCAAAG
Gm45437	CACACAGTGCCAGCAGAGT	GGTTCAGACCACGAGAGGA
